# Predictors of haemodynamic instability during the changeover of norepinephrine infusion pumps

**DOI:** 10.1186/s13613-016-0139-3

**Published:** 2016-04-22

**Authors:** Martin Cour, Thomas Bénet, Romain Hernu, Marie Simon, Thomas Baudry, Philippe Vanhems, Laurent Argaud

**Affiliations:** Service de Réanimation Médicale, Hospices Civils de Lyon, Hôpital Edouard Herriot, 69437 Lyon, France; Faculté de médecine Lyon-Est, Université Lyon 1, Université de Lyon, 69008 Lyon, France; Unité d’Hygiène, Epidémiologie et Prévention, Groupement Hospitalier Edouard Herriot, 69003 Lyon, France; Laboratoire des Pathogènes Emergents, Centre International de Recherche en Infectiologie (CIRI), INSERM U1111, CNRS UMR 5308, Université Lyon 1, Université de Lyon, Lyon, France

**Keywords:** Smart pumps, Septic shock, Norepinephrine, Changeover of vasoactive drugs, Drug safety

## Abstract

**Background:**

Changeovers of norepinephrine infusion pumps (CNIPs) frequently lead to haemodynamic instability. The aim of this study was to identify risk factors for haemodynamic instability associated with CNIP, independent of the method used to perform the relay.

**Methods:**

We performed a prospective study, in a university-affiliated intensive care unit. Over a 1-year period, all adult patients who had at least one CNIP were included. CNIPs were automatically performed using smart pumps, in accordance with a standardised protocol. CNIP-induced haemodynamic instability was defined as a variation in mean arterial pressure (MAP) >25 %. A multivariate mixed effects logistic regression was fitted to assess the factors associated with CNIP-induced haemodynamic instability.

**Results:**

From the 118 patients included in the study, 764 CNIPs were analysed. Most of the patients were treated with norepinephrine for septic shock of medical origin (*n* = 83, 70 %). Haemodynamic instability occurred 114 times (15 %) in 63 patients (53 %). Among the risk factors identified by the univariate analysis (age, heart rate, dose of norepinephrine infused, and change in the concentration of the vasoactive drug; *p* < 0.05), change in the norepinephrine concentration was the only independent risk factor for CNIP-induced haemodynamic instability identified in the multivariate analysis (adjusted OR 11.8, 95 % CI 7.2–19.5, *p* < 0.001).

**Conclusions:**

Changes in the norepinephrine concentration during CNIPs lead to a high risk of haemodynamic instability, while the clinical severity of patients, as well as the doses of norepinephrine, was not.

## Background

Norepinephrine is widely used as a first-line treatment to support blood pressure in critically ill patients [1]. Because of its narrow therapeutic range and very short half-life, all undesired changes in norepinephrine flow rates may lead to acute and potentially life-threatening changes in mean arterial pressure (MAP) [2]. In numerous countries, and almost all of Europe, norepinephrine is administered through high-precision electric syringe pumps [2–4]. As the volume of the syringes is limited (e.g. 60 ml for adults), changeovers of the norepinephrine infusion pumps (CNIPs) generally occur several times a day in an intensive care unit (ICU). Maintaining a constant flow rate during CNIP is challenging, and haemodynamic instability has been reported in up to one-third of vasoactive infusion pump relays [2–7].

Most of the research on CNIP has concentrated on determining the best method for performing these relays [2–8]. To date, it has been established that (1) the use of two syringe pumps (versus one), with or without an overlapping period, better ensures the continuity of the infusion, (2) the standardisation of the relay procedure limits haemodynamic incidents, and (3) the automation of the relay’s procedure, using smart pumps, further improves this routine care [2–8]. However, factors that are not linked to the methods, such as patient characteristics (including age, sex, type of shock, organ failures, and organ supports) and norepinephrine administration (e.g. concentration of the drug, flow rate, or dose), might also influence haemodynamic stability during CNIP.

Therefore, the aim of the study was to determine risk factors associated with CNIP-induced haemodynamic instability, regardless of the relay method, in order to identify new ways to further improve the safety of this procedure in the ICU.

## Methods

We performed a prospective, observational study, over a 1-year period in a university-affiliated 15-bed intensive care unit. This study was carried out during routine care with information of the patients or their relatives. Ethical approval was obtained from the ethics committee of the Hospices Civils de Lyon. This Institutional Review Board waived the need for consent given the nature of the study. The study was performed in compliance with the ethical standards detailed in the 1964 Declaration of Helsinki and according to French laws.

### Patients

All consecutive adult patients treated for shock with a continuous norepinephrine perfusion through a central venous catheter, and who had at least one CNIP, were included in the study. Invasive arterial blood pressure was continuously monitored (Intellivue MP monitor, Philips Medical Systems, the Netherlands) and recorded on a PC-based data acquisition system supported by IntelliSpace Critical Care and Anaesthesia (ICCA) software (Philips Medical System, the Netherlands). No change in haemodynamic management for cardiovascular dysfunction occurred in our ICU during the study period.

The following data were collected for each patient: gender, age, type of admission (i.e. medical or surgical), type of shock, organ failures according to the Sequential Organ Failure Assessment (SOFA) score, Simplified Acute Physiology Score II (SAPS II), number of CNIPs during ICU stay, length of stay in the ICU, and outcome.

### Norepinephrine administration

As previously described, norepinephrine was administered using a 60-ml capacity luer-lock syringe (BD Plastipack^®^, Octeville, France) and high-performance syringe pumps connected to a smart pump infusion workstation (Orchestra^®^ Base Intensive, Fresenius Kabi/Vial, Brezins, France), through a three-lumen central venous line [5]. The proximal lumen was always dedicated to the vasoactive drug infusion, and a low dead volume extension line with three-way stopcock (PES3101, Biocath^®^, Lissieu, France) was connected to this lumen in order to perform the CNIP. The initial dose of norepinephrine was determined empirically according to the severity of the shock state. Dose of norepinephrine was adjusted, as frequently as needed, to maintain haemodynamics.

### Preparation of norepinephrine infusion

At initiation of the treatment, dilution of norepinephrine (in 0.9 % sodium chloride) was left to the discretion of the physician to obtain one of the following concentrations: 0.17, 0.33, 0.50, 0.67, 0.83 and 1 mg ml^−1^. The purpose was to obtain an initial flow rate >2 ml h^−1^ to limit the time elapsed between the start-up of the infusion and the effective drug administration to the patient and <10 ml h^−1^ to limit the frequency of CNIP. Nurses were allowed to change the norepinephrine dilution at the time of the CNIP (i.e. when the volume of infusion was almost depleted) in order to maintain the flow rate in the predefined range (i.e. 2–10 ml h^−1^). Our standardised protocol was continuously available in a specific form in the ICU, and all the nurses received periodic refresher courses.

### Automated changeovers of norepinephrine infusion pumps

Changeovers of vasoactive drug’s syringes were performed up to 2 h before the end of the ongoing infusion. As previously described, the automated relay procedure that was provided by the smart pumps consisted of two associated channels, which infused the drug, one after the other, at the same dose [5]. Accordingly, when the concentration of the vasoactive drug changed in the course of the CNIP, the smart pump automatically modified the flow rate of the new infusion pump to maintain a constant dose of norepinephrine. Syringe pumps were connected to data acquisition system with automatic storage of flow rates and doses.

CNIP-induced haemodynamic instability was defined as a variation of MAP > 25 %, compared to the baseline, occurring within 30 min after the changeover. The treatments of the incidents were left to the appreciation of the physicians in charge of the patients and were not recorded.

### Statistical analysis

Categorical variables are expressed as counts and percentages and continuous variables as medians and interquartile ranges (IQRs), as appropriate. Comparisons of categorical variables were performed using two-sided Fisher’s exact tests. Continuous data were compared using the Mann–Whitney *U* test or Friedman’s test, as appropriate.

Factors associated with CNIP-induced haemodynamic instability were analysed by two-level mixed effects logistic regression (MELR) modelling. This model is derived from the generalised linear mixed model, and it allowed the estimation of both fixed effects (i.e. factors related to the syringe changeover) and random effects (i.e. related to the patient) [5, 7, 9]. The outcome was CNIP-related haemodynamic instability, as defined above. The two levels of the model were the characteristics of the changeover (Level 1) and of the patient (Level 2). The following explanatory data were related to the changeover: MAP, heart rate, organ supports, mechanical ventilation, renal replacement therapy, changeover order category (1st, 2nd–5th, >5th), norepinephrine concentration (mg ml^−1^), flow rate (ml h^−1^), doses (μg kg^−1^ min^−1^), and the change in the infused norepinephrine concentration. The following explanatory variables related to the patient were: gender, age category (<45 years, 45–59 years, 60–74 years, ≥75 years), SOFA score category (<10, 10–14, 15–19, ≥20), SAPS II category (<50, 50–64, 65–80, ≥80), type of admission (medical, surgical), and type of shock (septic, cardiogenic, haemorrhagic, other).

Covariates with a *p* < 0.15 after univariate MELR analysis were entered in the initial multivariate MELR model. MAP before CNIP was forced in the multivariate analysis. A stepwise backward process was then performed until all *p* values were <0.05, and models were compared using the Wald test. Odds ratios (ORs) are expressed followed by the 95 % confidence intervals (95 % CI).

 Stata 11 software (StataCorp. 2009. Stata Statistical Software: Release 11. College Station, TX: StataCorp LP) was used for analysis. Statistical significance was defined as a *p* value of <0.05. All tests were two-tailed.

## Results

Over the study period, we included 118 ICU patients, in whom 764 CNIPs were performed. As shown in Table 1, most of the patients were male (sex ratio of 2.4) and were admitted to the ICU for septic shock of medical origin (*n* = 83, 70 %). A majority of the patients (*n* = 63, 53 %) experienced at least one episode of haemodynamic instability during CNIP (Table 1). In these patients, both the SOFA score and the number of CNIP were significantly higher (*p* < 0.01) than in patients without CNIP-related incidents (Table 1).

**Table 1:** Patient characteristics

	Total (*n* = 118)	No incident^a^ (*n* = 55)	≥1 incident^a^ (*n* = 63)	*p*
Male sex^b^	83 (70)	39 (71)	44 (70)	>0.99
Age (years)^c^	63 (48–75)	63 (44–72)	63 (51–76)	0.35
Type of admission^b^				0.75
Medical	108 (92)	51 (93)	57 (90)	–
Surgical	10 (8)	4 (7)	6 (10)	–
Type of shock^b^				0.23
Septic	88 (75)	39 (71)	49 (78)	–
Cardiogenic	12 (10)	4 (7)	8 (13)	–
Haemorrhagic	10 (8)	6 (11)	4 (6)	–
Other	8 (7)	6 (11)	2 (3)	–
Fluid loading (litres)^c^	3.7 (2.4–5.4)	3.5 (2.2–5.4)	3.8 (2.6–5.4)	0.35
SOFA score^c^	12 (8–15)	11 (8–13)	13 (9–16)	<0.01
SAPS II^c^	59 (44–78)	53 (43–71)	61 (50–84)	0.06
ICU length of stay (days)^c^	8 (5–17)	6 (3–14)	11 (6–20)	<0.01
Number of CNIP^c^	4 (2–9)	2 (1–4)	8 (4–13)	<0.001
Survival^b^	67 (57)	36 (65)	31 (49)	0.07

*SOFA* Sequential Organ Failure Assessment, *SAPS II* Simplified Acute Physiology Score II

^a^Incident during changeover of norepinephrine syringe pumps (CNIP) was defined as a change in mean arterial pressure (MAP) >25 %

^b^Data are expressed as number (%) of patients

^c^Data are expressed as median values and IQRs

Characteristics of the 764 CNIP are reported in Table 2. A total of 114 (15 %) CNIP-related haemodynamic incidents were observed. Haemodynamic instability was significantly more frequent when patients received mechanical ventilation, when norepinephrine doses and flow rates were lower, and when there was a change in the concentration of norepinephrine (*p* < 0.05). Type and magnitude of incidents are depicted in Table 3. As expected, haemodynamic instability was an increase in MAP in 40/43 (93 %) CNIPs with decrease in norepinephrine concentration, whereas it was a drop in MAP in 13/15 (87 %) CNIPs with increase in norepinephrine concentration. No sustained ventricular arrhythmia and no cardiac arrest were directly attributed to a CNIP-related haemodynamic incident.

**Table 2:** Characteristics of the changeovers of norepinephrine syringe pumps

	Total (*n* = 764)	No incident^a^ (*n* = 650)	≥1 incident^a^ (*n* = 114)	*p*
Haemodynamics				
MAP (mmHg)^c^	67 (59–76)	67 (59–76)	67 (59–74)	0.70
Heart rate (bpm)^c^	107 (93–123)	107 (94–123)	105 (87–119)	0.06
Organ supports^b^				
Mechanical ventilation	729 (95)	616 (95)	113 (99)	0.04
Renal replacement therapy	470 (62)	397 (61)	73 (64)	0.60
Norepinephrine infusion^c^				
Flow rates (ml h^−1^)	5.4 (3.3–7.8)	5.7 (3.5–8.0)	4.0 (2.8–6.6)	0.001
Doses (μg kg^−1^ min^−1^)	0.6 (0.2–1.1)	0.6 (0.2–1.2)	0.4 (0.2–1.0)	<0.01
Norepinephrine concentration				
Concentration (mg ml^−1^)^c^	0.5 (0.2–0.5)	0.5 (0.25–0.5)	0.5 (0.5–0.5)	0.68
No change in concentration^b^	650 (85.1)	594 (91.4)	56 (49.1)	<0.0001
Change in concentration^b^	114 (14.9)	56 (8.6)	58 (50.9)	<0.0001
Decrease	68 (8.9)	25 (3.8)	43 (37.7)	<0.0001
Increase	46 (6.0)	31 (4.8)	15 (13.2)	<0.001
CNIP order^b^				0.95
First	118 (15)	101 (16)	17 (15)	–
2–5th	297 (39)	251 (39)	46 (40)	–
>5th	349 (46)	298 (46)	51 (45)	–

^a^Incident during changeover of norepinephrine syringe pumps (CNIP) was defined as a change in mean arterial pressure (MAP) >25 %

^b^Data are expressed as number (%) of patients

^c^Data are expressed as median values and IQRs

**Table 3:** Type and magnitude of haemodynamic incidents

	All CNIP with incidents^a^ (*n* = 114)	CNIP with no change in concentration (*n* = 56)	CNIP with increase in concentration (*n* = 15)	CNIP with decrease in concentration (*n* = 43)
Increase in MAP > 25 %^b^	83 (73)	41 (73)	2 (13)	40 (93)
Variation in MAP (%)^c^	33 (25–49)	31 (28–38)	27 (32–37)	36 (29–54)
Variation in MAP (mmHg)^c^	22 (17–32)	19 (15–25)	32 (27–37)	27 (20–35)
Decrease in MAP > 25 %^b^	31 (27)	15 (27)	13 (87)	3 (7)
Variation in MAP (%)^c^	33 (29–39)	33 (29–39)	33 (29–41)	26 (25–30)
Variation in MAP (mmHg)^c^	21 (19–28)	26 (20–30)	21 (19–31)	22 (17–23)

^a^Incident during changeover of norepinephrine syringe pumps (CNIP) was defined as a change in mean arterial pressure (MAP) >25 %

^b^Data are expressed as number (%) of patients

^c^Data are expressed as median values and IQRs

After univariate MELR analysis, age, heart rate, dose of norepinephrine, and change in norepinephrine concentration were significantly associated with haemodynamic incidents (Table 4). Gender, type of admission, type of shock, SAPS II score, SOFA score, MAP, mechanical ventilation, renal replacement therapy, norepinephrine infusion concentration and flow rates, and CNIP order were not linked with the risk of haemodynamic incident (data not shown). After multivariate MELR analysis, change in norepinephrine concentration was the only independent factor associated with an increased risk of haemodynamic incident (adjusted OR 11.8, 95 % CI 7.2–19.5, *p* < 0.001). Among CNIP with a change in norepinephrine concentration, hemodynamic instability was more likely to occur with low norepinephrine doses and when the drug concentration decreased (Table 5).

**Table 4:** Factors associated with haemodynamic incidents, univariate mixed effects logistic regression

	Crude OR (95 %CI)	*p*
Age category (years)		
<45	1.00 (Ref.)	–
45–59	2.3 (1.1–4.5)	0.02
60–74	1.5 (0.8–2.9)	0.25
≥75	2.0 (1.0–4.1)	0.05
Heart rate: per 10 bpm	0.9 (0.8–1.0)	0.04
Norepinephrine dose: per 1 μg kg^−1^ min^−1^	0.7 (0.5–0.9)	0.01
Change in concentration	11.8 (7.2–19.5)	<0.001

*OR* odds ratio, *CI* confidence intervals

## Discussion

In the present work, the change of the norepinephrine concentration during CNIP was the sole independent risk factor for blood pressure instability following this routine care, regardless of the method used for the norepinephrine syringe CNIP, which was automated and standardised. Contrary to popular belief, norepinephrine doses and flow rates, as well as the patient’s level of clinical severity, were not independently associated with CNIP-related haemodynamic instability. However, among changeovers with a change in norepinephrine concentrations, haemodynamic instability was more likely to occur with low dose of norepinephrine and when the drug concentration was decreased.

In this study, we observed haemodynamic instability in about 15 % of the CNIPs. This result agrees with the few studies published on this topic in which the frequency of haemodynamic incidents related to vasoactive infusion pump changeovers varied from 5 to 38 % [2–7, 10, 11]. Obviously, the frequency of these incidents depends on the definition of CNIP-induced haemodynamic instability, which is not consensual [2–7, 10, 11]. We chose to define haemodynamic instability as a relative change in MAP rather than an absolute change in MAP. Indeed, the risk with choosing an absolute value is the underestimation of CNIP-induced haemodynamic instability in patients with the lowest MAP. We defined haemodynamic instability as a CNIP-induced variation of MAP > 25 %. This threshold seemed clinically significant and corresponds to an absolute change of MAP of 15–20 mmHg for septic shock patients to obtain a therapeutic blood pressure target of around 70 mmHg. Because previous studies comparing methods of CNIP used similar norepinephrine concentrations in the two syringes, frequency of CNIP-related incidents may have been underestimated by comparison with “real life” [2–8, 10, 11]. Indeed, it is often necessary in clinical practice to change the concentration of the norepinephrine infusion throughout the administration of the drug. To the best of our knowledge, little data are available on changeover with changes in norepinephrine concentration. In the present work, while CNIP with changes in norepinephrine concentration represented almost 1/6 of the changeovers, they accounted for half of the haemodynamic incidents.

**Table 5:** Interaction between the norepinephrine dose and the risk of CNIP-induced haemodynamic instability based on changes in the norepinephrine concentration

	Overall (*n* = 764)		Dose < 0.5 μg kg^−1^ min^−1^ (*n* = 324)		Dose ≥ 0.5 μg kg^−1^ min^−1^ (*n* = 440)	
	Adjusted OR^a^ (95 % CI)	*p*	Adjusted OR^a^ (95 % CI)	*p*	Adjusted OR^a^ (95 % CI)	*p*
No change in concentration	1.00 (Ref.)	–	1.00 (Ref.)	–	1.00 (Ref.)	–
Decrease in concentration	20.7 (11.0–39.0)	<0.001	28.9 (13.5–61.8)	<0.001	7.8 (1.5–41.3)	<0.01
Increase in concentration	5.4 (2.7–10.9)	<0.001	33.2 (6.0–182.3)	<0.001	3.4 (1.4–8.1)	<0.01

*CNIP* Changeover of norepinephrine syringe pumps, *OR* odds ratio, *CI* confidence intervals

^a^After multivariate mixed effects logistic regression

We found that the only independent factor associated with CNIP-induced blood pressure instability was the change in the norepinephrine concentration. Contrary to what one might expect, the frequency of CNIP-related incidents was not independently associated with the norepinephrine doses infused into the patients and had no link to the severity of the shock. An important implication of this observation is that special care must be taken in all patients when performing CNIP, even in those with low doses of norepinephrine. Importantly, we took into account, in the most appropriate way, all of the usual factors known to alter the continuity of the norepinephrine infusion during CNIP (e.g. low flow rates, entrapped air in the perfusion line, vertical displacements of syringe pumps) [2, 12–14]. Above all, we used the safest available method to perform CNIP [5, 7, 8]. Indeed, both recent basic evidence and clinical evidence have suggested that the automation of a CNIP method without overlapping the perfusions (i.e. “quick change”), using smart pumps, better maintains the delivered norepinephrine doses and further improves blood pressure stability during the syringe changeovers [5, 7, 8].

Considering the number of haemodynamic incidents occurring when there is a change of norepinephrine concentration during CNIP, our study raises the problem of the use of several concentrations of norepinephrine in the ICU. We believe that the use of several dilutions of norepinephrine is of great clinical interest. Indeed, doses of norepinephrine may vary widely during the treatment of shock [15]. Thus, the use of a single dosage form of norepinephrine brings with it the possibility of administering the vasoactive drug at very low or very high flow rates. It is well known that small flow rates delay the delivery of a drug to the patient during treatment initiation, especially when the dead space of the infusion system is important [16–19]. It is reasonable to assume that such low flow rates play a role in altering the continuity of perfusion during CNIP. However, as flow rates were maintained at >2 ml h^−1^ in almost all patients at the time of the CNIP, our study does not answer whether low flow rates also alter the continuity of perfusion during CNIP. By contrast, high flow rates (e.g. >10 ml h^−1^) accelerate the emptying of the syringe and consequently increase the frequency of CNIP, which represents a high-risk period of haemodynamic instability [2–6, 10, 11].

Our results suggested that an automated “quick change” was probably not optimal for CNIP with changes in norepinephrine concentrations, particularly at low doses. Obviously, in the event of a change in the norepinephrine concentration during CNIP, the vasoactive drug solution occupying the dead space of the perfusion line is administered for some time at an undesired dose (depending on the drug concentration in the ending syringe and the flow rate of the new syringe pump). Consequently, this may induce a transient over- or underdose. For this reason, the number of CNIP with changes in norepinephrine concentrations should be limited as far as possible. We can hypothesise that the “double pumping” method, which provides an overlapping of the perfusions, might limit this inconvenience [3]. However, it is acknowledged that this process is time-consuming and not automatically provided by the current generation of smart pumps [3, 5]. Automated “quick change” might be easily improved by integrating the dead space of the perfusion line into the smart pumps’ algorithms. Future research is therefore required to address the important issue of safety during CNIP in the subgroup of changeovers with a change in norepinephrine concentration.

Whether CNIP-induced haemodynamic instability has an impact on clinical outcomes remains uncertain. Although our study was the largest on CNIP, it was not sufficiently powered to assess this important issue. It remains also unclear whether CNIP-induced increase in MAP, which occurred in the three quarters of the cases in our study, is less deleterious than CNIP-induced hypotension. This probably depends on both patients and types of shock (e.g. septic versus haemorrhagic) and on the magnitude of change in MAP. A final important question is still pending: how to manage the stressful CNIP-induced hemodynamic incidents? Further research is needed to answer these questions.

## Conclusions

Our results emphasise that the risk of haemodynamic incident during the CNIP is not linked to the severity of the shock or to the characteristics of the patients. Haemodynamic instability remains a high risk when the norepinephrine concentration is changed during CNIP. Further studies are needed to determine the best method for CNIP when a change in the norepinephrine concentration is required.

## Abbreviations

CNIPs: changeovers of the norepinephrine infusion pumps
ICU: intensive care unit
ICCA: IntelliSpace Critical Care and Anaesthesia
IQRs: interquartile ranges
MAP: mean arterial pressure
MELR: two-level mixed effects logistic regression
ORs: odds ratios
SAPS II: Simplified Acute Physiology Score II
SOFA: Sequential Organ Failure Assessment
